# Economic burden of malaria inpatients during National Malaria Elimination Programme: estimation of hospitalization cost and its inter-province variation

**DOI:** 10.1186/s12936-017-1934-5

**Published:** 2017-07-19

**Authors:** Shangfeng Tang, Da Feng, Ruoxi Wang, Bishwajit Ghose, Tao Hu, Lu Ji, Tailai Wu, Hang Fu, Yueying Huang, Zhanchun Feng

**Affiliations:** 1 School of Medicine and Health Management, Tongji Medical College, Huazhong University of Science & Technology, Wuhan, Hubei China; 2 Bureau of Disease Prevention and Control, National Health and Family, Beijing, China; 3 Cancer Center, Sun Yat-sen University, Guangzhou, Guangdong China

**Keywords:** Economic burden of malaria, Hospitalization cost, Elimination, Daily cost

## Abstract

**Background:**

Apart from its direct impact on public health and well-being, malaria had placed significant socioeconomic burden on both individuals and whole health systems. This study was conducted to investigate the hospitalization cost of malaria and explore the inter-province variation during the National Malaria Elimination Programme in China.

**Methods:**

Information on medical expenditure for malaria treatment was extracted from inpatient medical records in Henan, Hainan and Guangxi Province. The costs were adjusted to the price in 2014 and converted to USD (United States Dollars). Non-parametric and parametric methods were employed to estimate hospitalization costs and non-parametric bootstrap method was used for the comparison of hospitalization costs among sample provinces and to estimate the uncertainty of differences in inter-province hospitalization costs.

**Results:**

The hospitalization cost and daily cost of 426 malaria inpatients were 929.8 USD and 143.12 USD respectively. The average length of stay was 11.95 days. The highest cost of hospitalization services occurred in tertiary hospitals (956 USD per episode). Whereas the lowest ones occurred in internal departments (424 USD). Medications, laboratory tests and supportive resources for treatment were the most important components of hospitalization costs, respectively responsible for 45.31, 24.70, and 20.09% of the total hospitalization costs. The hospitalization cost per episode in Henan Province was significantly higher than that in Hainan an in Guangxi Province, with incremental costs of 713 USD (95% confidence interval 419.70, 942.50) and of 735.58 USD (95% CI 606.50, 878.00), respectively. The differences in the daily costs between Henan and Hainan along with Guangxi provinces were 75.33 USD (95% CI 40.33, 96.67) and 93.56 USD (95% CI 83.58, 105.28), respectively.

**Conclusions:**

Although the prevalence of malaria cases has considerably declined, the direct hospitalization costs of malaria in the household remain high and the inter-province variations need to be seriously considered in the formulation the further interventions regarding hospitalization cost control. This study suggests that economic risk protection mechanisms targeting at malaria inpatients should be redesigned. The drug price addition policy in public hospitals should be gradually reformed or abolished coupling with increasing government subsidies along with the charges for treatment services to reduce the hospitalization cost. The policy for cost control in the provincial hospitals should be implemented in comparison with the policy in other provinces, where the status of economic and geography are similar.

## Background

Malaria is generally associated with negative impact on the socioeconomic development of countries [1–4]. Underdevelopment countries, such as in western Africa, are suffering from most serious malaria-induced morbidity and mortality [5]. The distribution of per-capita GDP (gross domestic product) highlights a striking interaction between malaria and poverty in these countries [6]. Meanwhile, existing studies have also revealed that malaria placed great economic burden on both individuals and whole health systems. In southeast Nigeria, the non-recurrent provider cost reached up to $1857.15 for malaria hospitalization services per episode, while it only costs an average $23.20 at the household level [7]. Similar information has been reported in Zambia where the scale-up of malaria control greatly affected the health system at the facility level [8]. At the micro level, malaria directly threatens households due to loss of time and lower productivity [4], expenditure for malaria treatment [6], loss of household income [9], even early death [10]. Additionally, previous studies also demonstrated that demand of poor population for health care was sensitive to price [11], indicating that any incremental price in consumption might restrain the demand or incur catastrophic expenditure to poorer households [12]. Therefore, medical services may be delayed or refused, at the risk of causing natural transmission [13, 14].

In China, for the purpose of achieving malaria elimination by 2020, a mid-term evaluation of the National Malaria Elimination Programme was carried out in 2015. The evaluation revealed the remarkable progress achieved in the past decade. The number of malaria cases decreased from 7855 in 2010 to 3078 in 2014 [15], including 170 severe malaria cases with 25 deaths [16]. However, the imported malaria cases accounted for almost 98.1% of the total number in 2014, not only causing new threats to malaria elimination but also placing a significant economic burden on households [10]. Since the Global Fund to Fight Acquired Immune Deficiency Syndrome, Tuberculosis and Malaria completed in 2013, approximately 116 million USD had been disbursed for fighting malaria [17]. The Chinese government has continued to support malaria elimination since then [15]. In comparison with national expenditure estimates, data for the economic effects of malaria on households also need to be collected in order to improve the understanding of burdens on households and guide local health, social and policy interventions [3, 18].

Contrasting with the epidemiological studies of malaria [19], there is less evidence from China regarding the economic burden on households, particularly the incurred costs in medical services during the malaria elimination programme. There is only one systematic survey related to the economic burden of malaria, which involved 923 malaria cases (only 167 inpatients) in China. However, the economic burden of malaria inpatients deserves more attention considering that it is 4 times higher than in outpatients [10]. This topic has also attracted much research interest among authorities and scholars recently [20]. The costs for treating malaria patients estimated by previous studies vary largely among areas due to the variety of geographical location and socio-economic status [21, 22]. Therefore, for the purpose of investigating the hospitalization cost of malaria and revealing its inter-province variations in the elimination phase, this study was conducted in three provinces in China, which are geographically specific. Notably, treatment may be refused by the poor due to cost, with a knock-on effect on transmission and efforts for elimination, hence, the hospitalization cost is only referred to the cost occurring during hospitalization in an episode.

## Methods

### Study procedures and data collection

China is located beside the western Pacific Ocean, and it consists of 34 provincial-level administrative regions, with 56 ethnicities. Historically, Henan, Hainan and Guangxi Province were three high prevalence areas, where socioeconomic development had once been seriously influenced by malaria. In this case, these three provinces were selected as the study sites. Henan Province is located in central China. A total 1773 malaria cases with 12 deaths was reported from 2010 to 2014 in Henan. Hainan Island is located in the southernmost of China in the South China Sea. In history, Hainan province was a high prevalence endemic region, where 4422 falciparum malaria cases were reported during 2002–2012 [23], whereas a total of 122 malaria cases were reported from 2010 to 2014. Guangxi autonomous region, adjacent to Vietnam, consists of numerous ethnicities in the mountain area, with 1837 malaria cases reported from 2010 to 2014. Notably, the number of malaria cases sharply increased to 1251 cases in 2013, which accounted for 30.3% of the total 4128 cases in China [24]. Amongst the 1251 cases, the majority (1052, 84.09%) was reported from Shanglin county. More details are shown in Table 1.

**Table 1:** The incidence of each Plasmodium in three provinces (2010–2014)

Province	Plasmodium		*Plasmodium vivax*		*Plasmodium falciparum*		Unclassified	
	N	Incidence	N	Incidence	N	Incidence	N	Incidence
Henan	1773	0.3765	1115	0.2367	655	0.1391	3	0.0006
Guangxi	1837	0.7815	247	0.1051	1449	0.6165	141	0.0600
Hainan	122	0.2779	46	0.1048	34	0.0774	42	0.0957

The unit of the incidence is 1/100,000. The data was extracted from National Scientific Data Sharing Platform for Population and Health. http://cdc.ncmi.cn/Share/ky_sjml.jsp?id=784b16ae-76d4-48a3-9f83-a27591159f59&show=0

These study sites are less developed regions in China, where the malaria burden could not be ignored. Henan is an agricultural province with the largest population (94 million) in China. The GDP (gross domestic product) per capita was 6035 USD, ranking 23th of 31 provinces in 2014, similar with Hainan province (22th) and higher than Guangxi (28th). In terms of disposable household income (DHI), there were large gaps between urban and rural regions. This is particularly the case in Guangxi autonomous region, where the gap between rural and urban regions was the largest and DHI per capital was the lowest among three study sites. More details are shown in Table 2.

**Table 2:** The population and economic status of Henan, Hainan and Guangxi province

Characteristics	Henan	Hainan	Guangxi
Population (million)	94	9	48
Per capita GDP(USD)	6035	6234	5387
Per capita disposable household income (USD)	2555	2561	2292
Per capita disposable urban household income (USD)	3854	3661	4016
Per capita disposable rural household income (USD)	1622	1614	1414

The retrospective data from hospitals were collected from January 2015 to January 2016. By applying a two-stage cluster sampling method, four public hospitals designated for malaria treatment were purposely selected from each sampling province. All cases with a primary diagnosis of malaria and treated between 2010 and 2014 in the selected hospitals were collected for further analysis. One hospital in the provincial capital and three hospitals at the county level were chosen based on their workload regarding malaria treatment. A total of twelve hospitals including three hospitals at the provincial level and nine hospitals at the county level were selected. Finally, only five hospitals were included in this survey due to the insufficient inpatient cases in the other seven hospitals in last 5 years. Due to the lack of inpatient cases from 2010 to 2014, a few were retrieved from hospitals of Hainan, where no local falciparum malaria reported since 2010 [11, 25]. Hence, the inpatient data of another 5 years, that is from 2006 to 2010, were collected and the direct medical expenses with inflation were adjusted to the 2010 level using a series of Hainan health care CPIs (consumer price indexes).

As laboratory facilities were available at each hospital [26], every case diagnosed as malaria from 2010 to 2014 (Hainan: from 2006 to 2014) and confirmed by the Centre for Diseases Control and Prevention were included in this analysis. After obtaining a consent for using anonymized data from the sampling hospital authorities, the direct medical cost along with enrollment information on the first page of medical records of malaria inpatients was extracted from the hospital electronic database. However, due to the lack of hospital information system in two hospitals, 94 handwritten medical records accounting for 22.12% of the whole sample were copied and entered into Excel database by undergraduates from Tongji Medical College.

### Measures

All data used in this study were extracted from patient-level medical records. Individual or household direct medical costs related to malaria treatment were mainly the costs for medications, laboratory tests, and treatment [10, 27], which were already classified by hospital information system based on the patients’ consumption details during their hospitalization. This type of information was directly obtained from the first page of medical records. Referring to one similar study [28], the direct medical cost in this study was classified into six categories: (1) Medications: including western medications and traditional Chinese medications, such as antibiotics and supportive treatment medications. Notably, anti-malarials were excluded from this category since they are free-of-charge and publicly available. (2) Laboratory tests: including serum biochemistry, full blood count, electrocardiograph, X-ray and other supportive diagnosis. (3) Treatment supportive resources: including operation, intravenous fluids, blood transfusion and medical disposable equipment. (4) Sick bed expense. (5) Services: including the services for treatment and hospitalization in either intensive care units or general care departments. (6) Others: including any other item not included in the above categories. The expense obtained at recent 5 years were adjusted to the price in 2014 using a series of health care CPI reported in China statistical yearbooks (the CPI from 2011 to 2014 was 103.3, 102.9, 101.7, 101.5, respectively). The currency was converted to United States Dollar (USD) by employing an average exchange rate at 2014 (1 USD = 6.1428 RMB).

Coupled with direct hospitalization costs, the demographic and treatment information such as age, gender, hospital names, clinical departments, and hospitalization dates, was collected simultaneously. Some information was converted to new variables. The variable at the hospital level was generated and grouped two categories (secondary, tertiary) based on the online introduction of the hospital. According to hospital accreditation of China, the secondary hospital with more than 100 beds is regarded as a regional hospital, which mainly provides comprehensive medical services to community residents and undertakes some tasks of teaching and scientific research. The tertiary hospital has to set more than 400 beds, provide the specialized medical services to regional residents, and undertake the higher education and scientific research tasks.

### Statistical analysis

The data analysis followed the method reported in an article regarding the intra-country variation of costs for malaria treatment [29]. Compared to the parametric methods, non-parametric bootstrap methods which suit the materials with a small sample and do not follow a normal distribution were more suitable for estimating the differences in the incremental cost between two groups. In this circumstance, describe statistics, median analysis and non-parametric bootstrap were undertaken to calculate the direct hospitalization costs and the differences in direct costs and its components among three provinces.

Statistical Package for Social Sciences (SPSS version 13.0; Inc., USA) was employed for the statistical analysis. Descriptive statistics approaches were used to describe the sample characteristics while Shapiro–Wilk test was adopted for the normality test of the distribution of daily cost, hospitalization cost and the components. Before using a parametric method to conduct the midpoint analysis, Levene’s test was used to examine the homogeneity of variance among three provinces. Based on the skewed results of direct cost and each component, the variance analysis was inappropriate to test the differences in cost across different groups at a significant level. Therefore, the median value was calculated, and the differences in the median value across groups were tested using non-parametric methods. The significant level p = 0.05 was set to identify whether the null hypothesis was rejected or not. Besides, non-parametric bootstrap was implemented in R Version 3.3.1. For the purpose of calculating the standard error of the median differences without any assumptions of data distribution, the biases of median difference across provinces were computed through 5000 bootstrap replications and the 95% CI (confidence interval) was estimated through BCA (bias-corrected and accelerated) percentiles approximation that has been identified to have a better efficacy in previous studies.

## Results

### Simple characteristics of inpatients

A total of 425 records of malaria inpatients have been analysed in this study. Cases from Henan, Hainan, and Guangxi, respectively, accounted for 63.5, 14.6, 21.9% of the total sample. The mean age of the subjects was 38.88 years and most of them were male (96.2%). The average length of stay was 11.56 days. The majority of these records was extracted from tertiary hospitals (93.4%), and 58.4% of the subjects were treated in the infectious department while 30.4% of the subjects were treated in the internal department. Details are shown in Table 3.

**Table 3:** Characteristics and hospitalization costs of malaria inpatients (USD)

Characteristics	Inpatients (n)	Percentage (%)	Median of hospitalization cost (USD)	Median of daily cost (USD)
Age				
<30	101	23.8	898	150
30–39	117	27.5	977	153
40–49	140	32.9	915	136
>50	67	15.8	885	121
Gender				
Male	409	96.2	936	144
Female	16	3.8	741	127
Province^a,b^				
Henan	270	63.5	1195	166
Hainan	62	14.6	462	87
Guangxi	93	21.9	480	72
Hospital level^a^				
Secondary hospital	28	6.6	332	101
Tertiary hospital	397	93.4	956	145
Clinic departments^a,b^				
Infectious unit	248	58.4	1188	160
Internal department	129	30.4	424	79
Other department	48	11.3	1409	168
Length of stay^a^				193
<5	89	20.9	426	25
5–7	137	32.2	898	150
8–10	104	24.5	977	153
>10	95	22.4	915	136

^a^Comparisons of sum hospitalization cost among subgroups tested through Kruskal–Wallis H test, p < 0.05

^b^Comparisons of daily cost among subgroups tested through Kruskal–Wallis H test, p < 0.05

### Hospitalization costs of malaria inpatients

Due to the skewed distribution of hospitalization and daily costs, the median cost of each subgroup was presented and tested by the non-parametric method. The results presented in Table 3 are in the price of 2014. The hospitalization cost and daily cost of the inpatient treatment in one episode were 930 USD and 143 USD, respectively. Remarkable differences in the hospitalization cost and daily cost were observed among provinces, and clinical department. No difference was found among subgroups of age and gender. The median cost of hospitalization services in tertiary hospitals was 956 USD per episode, which is approximately triple of that in secondary hospitals (332 USD). No difference in daily cost was observed. The hospitalization cost and daily cost in the internal department were the lowest with 424 USD and 79 USD per episode, compared with those in the infectious unit (1188, 160 USD, respectively) and other departments, including intensive care unit (1409, 168 USD).

**Fig. 1:**
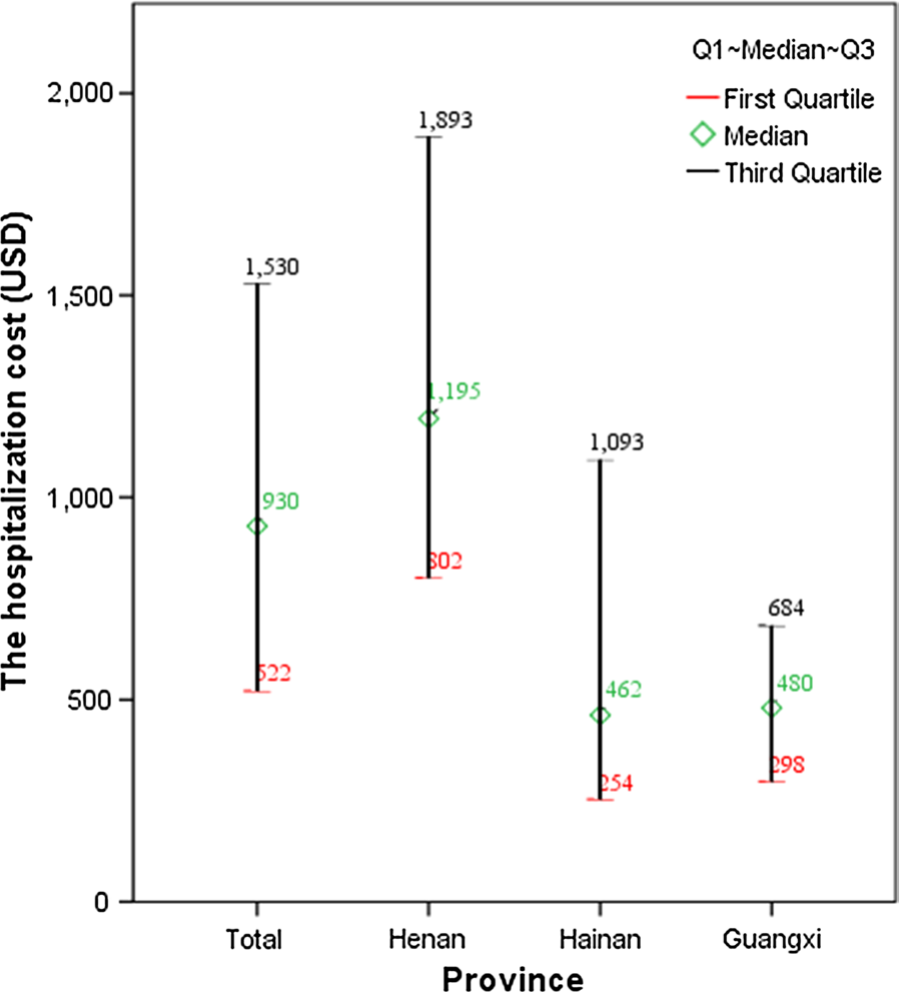
The costs and ranges of hospitalization by province (USD)

**Fig. 2:**
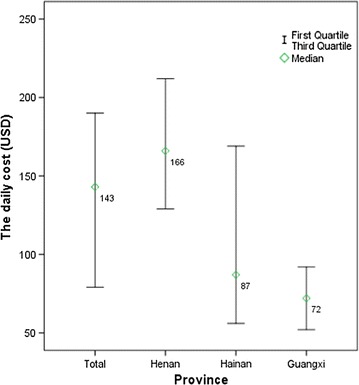
The costs and ranges of hospitalization per day by provinces (USD)

Notably, as shown in Fig. 1, the hospitalization cost in Henan province was the highest with 1195 USD per episode. This was approximately twice as much as that in Hainan (462 USD) or Guangxi provinces (480 USD). Referring to the hospitalization cost, a similar trend of the daily cost among three provinces was presented in Fig. 2. The daily cost in Henan province was the highest (166 USD) compared with that in Hainan (87 USD) and Guangxi provinces (72 USD). The first quartile value and median value of the hospitalization cost in Henan Province was similar with the corresponding data in Guangxi Province, so as the daily cost in these two provinces. However, the range of these two types of cost in Hainan ($254–1093 and $56–169, respectively) is much bigger than that in Guangxi ($298–684, $52–92, respectively). It may be explained by the fact that the different strain of the plasmodium parasites and the severity of complications in this area requires different therapies and prescription behaviours, which leads to variations in cost.

### The cost of hospitalization components

The median cost of medications and laboratory tests per episode was 421 USD and 333 USD. The median cost of supportive resources, sickbed and services was relatively low, respectively accounting for 56 USD, 38 USD and 34 USD. The hospitalization costs and costs for each component in Henan were generally higher than those in Hainan and Guangxi province. Compared with Guangxi, Hainan had higher medication (608 USD) and lower treatment costs (175 USD). More details of the cost of hospitalization components among three provinces are shown in Table 4.

**Table 4:** The costs (ranges) of each item in different provinces (USD)

Admission cost items	Total	Henan	Hainan	Guangxi
	Median (Q1, Q3)	Median (Q1, Q3)	Median (Q1, Q3)	Median (Q1, Q3)
Medications	421 (202, 846)	608 (368, 1052)	215 (94, 458)	141 (48, 275)
Laboratory tests	333 (123, 333)	406 (303, 566)	175 (98, 333)	230 (161, 288)
Supportive resources	56 (31, 117)	62 (35, 126)	26 (9, 78)	59 (34, 92)
Sickbed	38 (20, 63)	45 (32, 77)	20 (13, 65)	20 (13, 25)
Services	34(14, 62)	44 (30, 80)	17 (7, 57)	13 (10, 16)
Other	4 (2, 17)	11 (3, 20)	2 (1, 4)	2 (0, 3)

*Q1* the first quartile, *Q3* the third quartile

### Structure of hospitalization cost

**Fig. 3:**
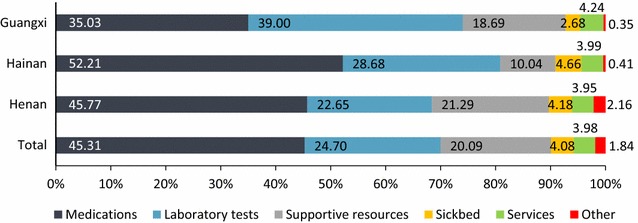
The structure of hospitalization cost among study provinces (%)

As shown in Fig. 3, medications, laboratory tests and supportive resources for treatment are the most important components of the total hospital cost, responsible for 45.31, 24.70, and 20.09% of the total cost. However, the cost of services for treatment and hospitalization care only accounted for 3.98%, with a range between 3.95 and 4.24% among three study provinces. The structure of hospitalization cost in Henan was similar with that in overall level, and the proportions of medications and laboratory tests in Henan were 45.77, 22.65%, respectively. In Hainan province, the medications cost was responsible for 52.21% of the hospitalization cost, whereas the proportion of the cost for treatment including sick bed, services and other cost was just 19.11%. Meanwhile, Guangxi with the lowest proportion of medications (35.03%) seemed to be the most reasonable in structure of hospitalization cost, but the cost of laboratory tests accounted for 39.00% of the hospitalization cost which was worthy more attention.

### Differences in hospitalization cost at the provincial level

As shown in Table 5, the differences of the costs in Henan–Hainan and of those in Henan–Guangxi were statistically significant. The hospitalization cost in Henan was significantly higher than those in Hainan and Guangxi provinces, with differences of 713 USD (95% CI 419.70, 942.50) and of 735.58 USD (95% CI 606.50, 878.00), respectively. Besides, the differences in the daily costs in Henan compared with that in Hainan and Guangxi provinces were 75.33 USD (95% CI 40.33, 96.67) and 93.56 USD (95% CI 83.58, 105.28), respectively. However, no significant difference in hospitalization and daily costs between Hainan and Guangxi (23.05 USD, 95% CI −152.32, 275.18; 18.32 USD, 95% CI −2.12, 53.12) was observed.

**Table 5:** Differences of daily cost, hospitalization cost and its components among provinces (USD)

Hospitalization costs	Median difference of Henan–Hainan (95% CI)	Median difference of Henan–Guangxi (95% CI)	Median difference of Hainan–Guangxi (95% CI)
Hospitalization cost	732.67	714.91	−17.76
Bootstrap	713.40 (419.7, 942.5)	735.58 (606.50, 878.00)	23.05 (−152.32, 275.18)
Daily cost	78.46	93.52	15.06
Bootstrap	75.33 (40.33, 96.67)	93.56 (83.58, 105.28)	18.32 (−2.12, 53.12)
Medications	393.31	467.74	74.43
Bootstrap	391.09 (296.60, 483.80)	457.17 (404.80, 553.5)	66.97 (−0.60, 173.19)
Laboratory tests	231.07	176.2	−54.87
Bootstrap	226.15 (181.30, 272.20)	179.23 (139.3, 210.8)	−46.17 (−104.29, −8.56)
Supportive resources	36.74	3.5	−33.24
Bootstrap	35.15 (21.18, 49.83)	3.82 (−10.37, 20.64)	−31.18 (−47.00, −14.70)
Sickbed	24.99	25.74	0.74
Bootstrap	24.84 (10.62, 31.17)	26.87 (22.34, 28.78)	2.07 (−5.15, 9.22)
Services	26.65	31.16	4.51
Bootstrap	25.47 (15.52, 35.77)	31.71 (27.28, 37.25	6.28 (−1.22, 16.48)
Other	9.41	9.27	−0.15
Bootstrap	9.30 (7.11, 11.20)	9.36 (6.09, 10.92)	0.06 (−0.94, 0.64)

Bootstrap was based on 5000 bootstrap replications

In terms of the components of hospitalization cost, the significant higher hospitalization cost in Henan, compared with those in Hainan, was highly associated with the incremental costs of medications (391.09 USD, 95% CI 296.60, 483.80), laboratory tests (226.15 USD, 95% CI 181.30, 272.20). Meanwhile, the incremental costs of treatment sick bed, and health services also positively contributed to the differences in hospitalization costs between Henan and Hainan.

Similar to the difference in hospitalization costs between Henan and Hainan, the incremental costs of medications (457.17 USD, 95% CI 404.80, 553.50) and laboratory tests (179.23 USD, 95% CI 139.30, 210.80) played the most important role in the significant higher hospitalization cost in Henan when compared with that in Guangxi. The cost of supportive resources (3.82 USD, 95% CI −10.37, 20.64) in Guangxi was similar with that in Henan, and both were higher than that in Hainan.

Although the direct hospitalization cost in Hainan seemed to be similar with that in Guangxi, the observational offset effect between the incremental medication costs (66.97 USD, 95% CI −0.60, 173.19) and declining costs associated with laboratory tests (−46.17 USD, 95% CI −104.29, −8.56) and supportive resources for treatment (−31.18, 95% CI −47.00, −14.70) were presented.

## Discussion

There were several limitations in this study. First, some important determinants of cost at the patient level, such as whether the health insurance released or induced the hospitalization cost or not, the severity of co-morbidities, different species of the Plasmodium parasites and the complications of patients, were not included in this study due to the lack of data. Second, along with the decrease of malaria cases in China, only 425 inpatients mainly from three provincial hospitals were collected to analyse. Simultaneously, since the cluster sampling method was employed to obtain the inpatients’ information at hospitals, the percentage of malaria inpatients among three provinces may not reflect the relative incidence of malaria in these three provinces, rather, the relative incidence of malaria inpatients in provincial hospitals. The comparisons by province in the study were not designed to really compare provinces, the sample cases may also not be representative to the study province rather than a provincial hospital for malaria treatment. Third, more evidence is needed to identify whether or not the hospital designed for malaria treatment will lead to an increase in the total hospitalization cost.

The hospitalization cost of malaria treatment needs to be controlled along with malaria elimination. This study indicated that the direct hospitalization cost of malaria treatment in China was seemingly higher than that in other countries. In Manaus of Brazil, the direct medical cost of 211.64 USD (95% CI 191.03, 229.82) per malaria inpatient at the price of 2011 [30] was reported in a similar study. It accounted for only 24% of 875.55 USD (the price adjusted at 2011) in this study. However, its GDP was higher than the average level of this study sites. One possible reason for the difference between China and Brazil may be due to the difference in malaria inpatient numbers. It would be expected that in areas with high malaria burdens, the per inpatient cost would be lower as fixed costs are spread over a larger number of patients. Meanwhile, another evidence showed that the cost for hospitalization services in China was much higher than that in Thailand [28]. Besides, the hospitalization cost was almost twofold compared to that in a recent study in China (2587 RMB, which adjusted and converted to 409.14 USD according to the value of money in 2011) [10]. Although Henan province was enrolled in both studies, it may be explained by the fact that all hospitalized malaria cases in recent 5 years were retrieved from a tertiary hospital in Henan where the average cost reached 1195 USD in this study.

Although the prevalence of malaria gradually declined during the Malaria Elimination Programme, the economic burden incurred by imported cases are worth more attention, especially for the one who had to seek hospitalized services in Henan province. The hospitalization cost was responsible for approximately 37.67% of the disposable household income on average. However, this proportion accounts around 60% in rural areas. According to a series of studies, the major concern was that if the out of the pocket of expenditure goes beyond 40% of household income, it may easily lead to catastrophic situations [31–33]. In China, there was universal coverage of basic medical insurance such as the New Rural Cooperative Medical Insurance Scheme for rural residents and the Urban Residents Medical Insurance Scheme. However, the practical proportion of reimbursement was around 55% at secondary hospitals and was even lower at tertiary hospitals. Therefore, from the perspective of the comparison between average hospitalization cost and the aforementioned average income, the rude estimation of out-of-pocket payment implied that it might be catastrophic for rural households. Simultaneously, it was also confirmed by the staff of CDC that if malaria patient has to seek hospitalization services, their expenditure of medical care will account for the majority of family income in this year. Additionally, a prior study in China showed that the indirect costs were higher than direct costs [10]. The length of stay is another representative indicator of indirect costs. It also tends to be longer than that in Zambia [8] and Britain [34].

The high hospitalization cost might be explained by shrinking the line of malaria treatment. Wide coverage of anti-malarial drugs was regarded as uneconomical measures in China, the line of malaria treatment was shrinking along with the elimination of malaria cases. Recent studies showed that township hospitals or community health centres took major responsibility on malaria surveillance [35, 36]. However, the majority of patients have to convert to secondary or tertiary hospitals seeking hospitalization services when they were suffering from malaria. This was consistent with another study revealing that patients had to either bypass the township hospitals or community health centres or follow hospital referrals due to lack of treatment facilities at the frontline level [31]. The insufficient stock of the anti-malarial drugs in township hospitals and lower level of knowledge of physicians in malaria eliminated areas might be the two most important factors pushing patients to seek services from tertiary hospitals [37]. Hence, the high hospitalization cost occurred coupling with the higher charging policy of tertiary hospitals.

In response to the treatment of malaria, one tertiary hospital in each provincial capital of study sites was designated as the malaria-treatment hospital since they have advanced facilities for treatment. The majority of falciparum malaria cases were detected and then referred to the designated hospital. Although no malaria-caused death in was observed in these study designated treatment hospitals during this period, the costs of seeking hospitalized services were high without any consideration of indirect cost. This phenomenon was particularly evident in Henan province. On the one hand, this study suggests that the economic risk protection mechanisms directing at malaria should be redesigned by the medical insurance schedule. On the other hand, the health management system at the provincial level needs to function more effectively [7] and to control hospitalization costs referring to other provinces where the economic and geography status is similar. Interestingly, the average cost occurred in internal departments was far less than that occurred in other departments where the cases accounted for 30.4% of total sample cases. It may be explained by the fact that patients with uncomplicated malaria cases may likely to be admitted by physicians and the severe malaria cases may often be found in other departments, such as ICU and the Department of Infectious Diseases.

Another finding of this study was that the hospitalization cost was mainly driven by medications, laboratory tests and supportive resources for treatment. This may be explained by the common benefits chasing behaviour in the hospital. Besides, according to the policy of China, anti-malarial medications are free of charge for the public. However, it does not mean that medications supported treatment are free of cost. In this study, the medication costs were too high to require more governmental supervision. On the contrary, the cost for service only accounted for approximately 4% of the total cost, the price of service need to be considered to accurately reflect the value of professional medical services for malaria treatment along with the hospitalization cost control. This study suggests that the government should gradually reform or cancel Drugs Price Addition Policy in public hospitals coupling with increasing government subsidies along with the charges in treatment services.

## Conclusions

Although the prevalence of malaria cases has considerably declined, the direct hospitalization costs of malaria to households remain high. It may likely be catastrophic to rural households in China, for the ones attacked by malaria and have to seek hospitalized services. Statistical differences in malaria hospitalization costs among the study sites were observed. The highest hospitalization cost in Henan province is worth more attention from health authorities for further intervention. Besides, shrinking the line of malaria treatment may contribute to the higher hospitalization cost, which mainly consists of the costs of medications, laboratory test and supportive resources for treatment. Additionally, this study suggests that economic risk protection mechanisms targeting for malaria inpatients should be designed. In order to stimulate the public hospitals’ initiative to provide the health services, the Chinese government has implemented a drug price addition policy, which allowed addition of 15% of drug cost price as hospital’s profit. In this study, the drugs price addition policy in public hospitals should be gradually reformed or canceled coupling with increasing government subsidies and the charges for treatment services, in order to reduce the hospitalization cost. The policy for cost control on the provincial hospitals should be implemented in comparison with others where the economic and geography status is similar.

## Abbreviations

USD: United States Dollars
GDP: gross domestic product
CI: confidence interval
DHI: disposable household income
